# Hematologic immune-related adverse events in skin cancer patients treated with immune checkpoint inhibitors: a case series

**DOI:** 10.3389/fphar.2025.1717727

**Published:** 2026-01-06

**Authors:** Rosa Falcone, Alessandra Frezzolini, Emanuele Bruni, Sofia Verkhovskaia, Maria Luigia Carbone, Francesca Romana Di Pietro, Paolo Chesi, Gabriele Piesco, Maria Cantonetti, Paolo Marchetti, Federica De Galitiis, Cristina Maria Failla

**Affiliations:** 1 Department of Oncology and Dermato-Oncology, IDI-IRCCS, Rome, Italy; 2 Laboratory of Immunology and Allergology, IDI-IRCCS, Rome, Italy; 3 Departmental Faculty of Medicine and Surgery, UniCamillus-Saint Camillus International University of Health Sciences, Rome, Italy; 4 Clinical Trial Center, Istituto Dermopatico dell’Immacolata-Istituto di Ricovero e Cura a Carattere Scientifico, IDI-IRCCS, Rome, Italy; 5 Experimental Immunology Laboratory, IDI-IRCCS, Rome, Italy

**Keywords:** anemia, hematologic toxicity, immunotherapy, melanoma, neutropenia

## Abstract

**Background:**

Immune checkpoint inhibitors (ICIs) are the gold standard therapy for cutaneous melanoma and are also used effectively in treating other types of skin cancer. Hematologic toxicities are rare, but potentially a serious and life-threatening side effect of ICIs. Clinical and biological biomarkers able to predict these events have been poorly explored and have not yet been identified.

**Case presentation:**

We present four cases of hematologic toxicity in melanoma patients and one case in a patient with cutaneous squamous cell carcinoma, all of which arose during treatment with ICIs in an adjuvant or metastatic setting. Hemolytic anemia was the most frequent event; neutropenia with agranulocytosis happened in one case and was fatal. ICI treatment was discontinued in all five cases and was never restarted. Two prevalent features were male sex and older age (>70 years old). These events were independent of the response to ICIs. Indeed, they occurred in a patient who progressed during treatment and in patients who responded completely to therapy. Previous diarrhea due to ICIs (patients 1, 2, and 5), asthenia (patients 3 and 4), and a sudden increase in lactate dehydrogenase levels despite the absence of disease progression (patients 2, 3, 4, and 5) might be warning signs of subsequent hematologic irAEs.

**Conclusion:**

Our study underscores the rarity and potential severity of hematologic toxicities, underlining the need for heightened clinician awareness and the incorporation of hematologic guidance into oncologic practice. Although predictive biomarkers remain unvalidated, monitoring immune cell subsets or recognizing warning signals early on may facilitate diagnosis and improve prognosis.

## 1 Introduction

Immune checkpoint inhibitors (ICIs) have transformed the treatment of patients with early- and late-stage melanoma by improving clinical and radiological responses and extending overall survival (Carlino et al., 2021). Despite their efficacy, ICIs can induce immune-related adverse events (irAEs), which are autoimmune toxicities that can affect any organ, including the skin, colon, liver, lungs, heart, endocrine glands, and bone marrow (Darnell et al., 2020). Hematologic toxicities are rare irAEs induced by ICIs, either as monotherapy or in combination. Currently, descriptions of these events are limited to case reports or retrospective analyses (Davis et al., 2019; Delanoy et al., 2019; Kroll et al., 2022). Due to the rarity of hematologic toxicities and the difficulty of diagnosing them, it is challenging to estimate their incidence. However, some authors suggest frequencies of 3.6% for all grades of toxicity and 0.7% for grades 3–4 (Michot et al., 2019). Toxicities of all grades were found to be higher with anti–programmed cell death-1 (PD-1) (4.1%) (nivolumab or pembrolizumab) or anti–programmed cell death-ligand 1 (PD-L1) (4.7%) than with anti–cytotoxic T-lymphocyte–associated protein 4 (CTLA4) (0.5%) (ipilimumab) (Michot et al., 2019). The mortality rate for high-grade hematologic irAEs is around 14%. Due to their rarity and the poor understanding of their clinical presentation and spectrum of events, they are not always recognized and promptly managed. The range of clinical manifestations includes immune thrombocytopenia, aplastic anemia, pancytopenia, neutropenia, autoimmune hemolytic anemia (AIHA), bicytopenia, pure red cell aplasia, hemophagocytic lymphohistiocytosis, and acquired hemophilia A (Kramer et al., 2021). The time to onset varies; half of hematologic irAEs appear within the first 10 weeks after ICIs are initiated, with most resolving within one to 2 months (Delanoy et al., 2019). Regarding the anti-PD-1 antibody cemiplimab, which is used to treat skin squamous cell carcinoma, a study reported only neutropenia occurrence (Di Lorenzo et al., 2024). Although rare, the widespread use of ICIs to treat various cancer types increases the likelihood of encountering hematologic irAEs (Keam et al., 2024). Therapeutic strategies include the use of steroids, intravenous immunoglobulins, rituximab, blood component transfusions, growth factor support, and immunosuppressants (Barnes et al., 2021; Omar et al., 2020). Early consultation with a hematologist is advised for diagnosis and management. A bone marrow examination should be considered to rule out other causes of pancytopenia, such as marrow infiltration, secondary myelodysplastic syndrome, or aplastic anemia.

Currently, there are no identified predisposing risk factors or prognostic biomarkers that can prevent patients from developing such toxicities.

## 2 Case series

We present five cases of skin cancer patients (Table 1) we observed in a period of 3 years who developed grade 2 to 4 hematologic toxicity to ICI therapy, as graded according to the Common Terminology Criteria for Adverse Events (CTCAE) v5.0. Four patients had cutaneous or mucosal melanomas, and one patient had cutaneous squamous cell carcinoma. Data regarding comorbidities, medications, relapse, follow-up, and rechallenge with ICIs are presented in Table 2. Data on available hematologic elements are reported in Table 3.

**TABLE 1:** Clinical characteristics of the patients.

Patient/sex	Age (years)	Tumor mutation -PD-L1 expression	ICI	Setting	Hematologic event – grading^a^	Time to event (mo)	Best response to ICI	Death
1M	73	BRAF wt c-KIT wt N-RAS n.a. PD-L1 < 1	Nivolumab + ipilimumab	1^st^ L	Agranulocytosis G4	3.5	PD	Y
2M	74	BRAF wt N-RAS Q61X PD-L1 < 1	Ipilimumab	2^nd^ L	AIHA G4	0.5	PR	N
3M	84	BRAF wt N-RAS Q61X PD-L1 n.a.	Nivolumab	adj	AIHA G4	7	NED	N
4F	78	BRAF K601E PD-L1 > 1	Pembrolizumab	adj	AIHA G2	1.5	NED	N
5M	78	n.a.	Cemiplimab	1^st^ L	MDS G4	4	CR	Y

M: male, F: female, ICI: checkpoint inhibitor immunotherapy, L: line of treatment, G: grading, AIHA: autoimmune hemolytic anemia, MDS: myelodysplastic syndrome, adj: adjuvant, NED: no evidence of disease, PD: progressive disease, PR: partial response, CR: complete response, mo: months, Y: yes, N: no; n.a.: not available, BRAF: B-Raf proto-oncogene, N-RAS: Neuroblastoma RAS, Viral (V-Ras), wt: wild type, PD-L1: programmed cell death-ligand 1.

^a^ Toxicities were graded according to CTCAE v5.0 criteria.

**TABLE 2:** Clinical data on patient clinical situations and outcomes.

Patient	Comorbidies	Concomitant drugs	Response to irAE treatments	Last f-up	Relapse - progression	Current status^a^	Rechallenge
1	None	None	No	11/2024 (death)	Yes	-	No
2	-Arterial hypertension -Benign prostatic hyperplasia	Valsartan Dutasteride	Yes	11/2025	Yes	Palliative	No
3	-Arterial hypertension -Benign prostatic hyperplasia	Irbesartan Tamsulosin	Yes	11/2024	No	Disease-free	No
4	History of multiple drug and food allergies	None	No	11/2025	Yes	Palliative	No
5	-Arterial hypertension -Chronic renal failure -History of multiple myeloma	Amlodipine	No	5/2025 (death)	No	-	No

F-UP: follow up, irAE: immune-related adverse events.

^a^ Last evaluation: Patient 2: CT, on November 2025, Patient 3: CT, on November 2024, Patient 4: CT, on October 2025.

**TABLE 3:** Transfusion details and outcome.

Patient	Transfusion number of unit	Reactions to transfusion	Threshold	Steroid treatment^a^
1	None	NA	8	Concomitant
2	7	None	7	After
3	3	None	7	Concomitant
4	None	NA	8	After
5	None	NA	8	Concomitant

NA: not applicable.

^a^ 1 mg/kg. Concomitant: the patient was under steroid treatment at the time of the irAE.

After: the patient received steroid treatment after the diagnosis of the irAE.

### 2.1 Case 1

#### 2.1.1 Patient information and clinical findings

The first case was a 73-year-old man with metastatic mucosal melanoma of the anorectal junction, which was diagnosed in July 2024. The patient was in good clinical condition and had no comorbidities in his medical history. The melanoma was BRAF wild-type and negative for c-KIT and PD-L1. Due to the extent of the disease, which included multiple bilateral iliac, obturator, and presacral lymph nodes, as well as symptoms, the patient began palliative radiotherapy on the primary tumor and systemic immunotherapy with ipilimumab (3 mg/kg), plus nivolumab (1 mg/kg).

#### 2.1.2 Timeline

After the first cycle of immunotherapy, the patient developed grade 3 diarrhea, which went into remission after receiving a 1 mg/kg dose of steroids. Due to toxicity and limited treatment options, ipilimumab was discontinued and nivolumab was administered for two additional cycles without the appearance of other symptoms. However, a computed tomography (CT) scan performed in October 2024 revealed significant disease progression in the liver, lungs, and lymph nodes. Lactate dehydrogenase (LDH) increased to 486 U/L. The patient was admitted to the hospital for a liver biopsy. On the first day, the patient had grade 4 febrile neutropenia: 2.0 × 10^3^ μL white blood cells, 0.01 × 10^3^ μL neutrophils, 1.75 × 10^3^ μL lymphocytes, and 0.05 × 10^3^ μL monocytes. His hemoglobin and platelet levels were within normal values, and his C-reactive protein (CRP) level was 162.9 mg/L.

#### 2.1.3 Outcomes and follow-up

Over the next month, the patient received meropenem, piperacillin/tazobactam, high-dose intravenous steroids, an antimycotic, and daily granulocyte colony-stimulating factor, but there was no improvement. A bone marrow biopsy showed aplasia limited to the myeloid cells. The patient became depressed and decided to go home against medical advice. The patient died the day after, just 4 months after the melanoma diagnosis.

### 2.2 Case 2

#### 2.2.1 Patient information and clinical findings

The second case was a 74-year-old man with a history of multiple thin skin melanomas. In 2015, he underwent a liver resection for a single metastatic melanoma lesion. Two years later, the disease progressed to his lungs and lymph nodes. He received pembrolizumab therapy for 3 years, until 2020, when it was discontinued due to a complete response and grade 2 diarrhea.

#### 2.2.2 Timeline

Pembrolizumab was restarted in 2023 due to histologically confirmed progressive disease. Molecular analysis revealed an absence of the BRAF V600 mutation and the presence of the N-RAS Q61X mutation. The patient received pembrolizumab treatment for approximately 10 months. In August 2024, the patient presented with anemia (grade 2), with a hemoglobin level of 8.5 g/dL, down from an initial level of 11.2 g/dL. The occult blood test was negative, and the patient refused an endoscopic evaluation. Hemoglobin values remained around 9 g/dL for the following months. In October 2024, further progression occurred, and a second-line therapy with ipilimumab was initiated. A few days after the first cycle of ipilimumab, the patient was admitted to the emergency room due to severe asthenia and rapid clinical deterioration. Blood tests revealed a hemoglobin level of 4.06 g/dL (grade 4 anemia), a total bilirubin level of 6.5 mg/dL, a CRP level of 138 mg/L, and a positive procalcitonin level of 10.7 ng/mL. Coagulation tests were within the normal range, but haptoglobin levels were low, and LDH and ferritin levels were high (1348 U/L and 2,252 ng/mL, respectively). A CT scan performed in November 2024 did not show any progressive disease.

The Coombs test was positive. Gastroscopy and colonoscopy were negative.

#### 2.2.3 Outcomes and follow-up

The patient received seven bags of blood transfusions, antibiotic therapy, and 1 mg/kg of steroids intravenously. Fifteen days later, the patient was discharged with improved anemia (Hb 9.9 g/dL). CRP and procalcitonin were negative, and bilirubin was within normal ranges. There was an indication for tapering steroid therapy. Three months after the single dose of ipilimumab, the patient is still alive and progressing. Due to oligo-progression, the patient received local treatment, including surgery for cutaneous metastasis and radiotherapy for a lymph node. Thus far, he has not restarted any systemic therapy.

For these first two patients only, blood samples were available at the beginning of ICI treatment and at the onset of hematologic irAE. Thus, an analysis of the circulating immune cells was performed using multicolor flow cytometry to assess the frequency of total circulating T cells (CD3^+^), B cells (CD19^+^) and natural killer (NK) cells (CD16^+^CD56^+^CD3-), as well as the relative amount of CD8^+^ and CD4^+^ T cells in the patients' whole blood. The flow cytometry method, flow diagrams, gating strategies, and quantification results are described in Supplementary Figure S1.

**FIGURE 1:**
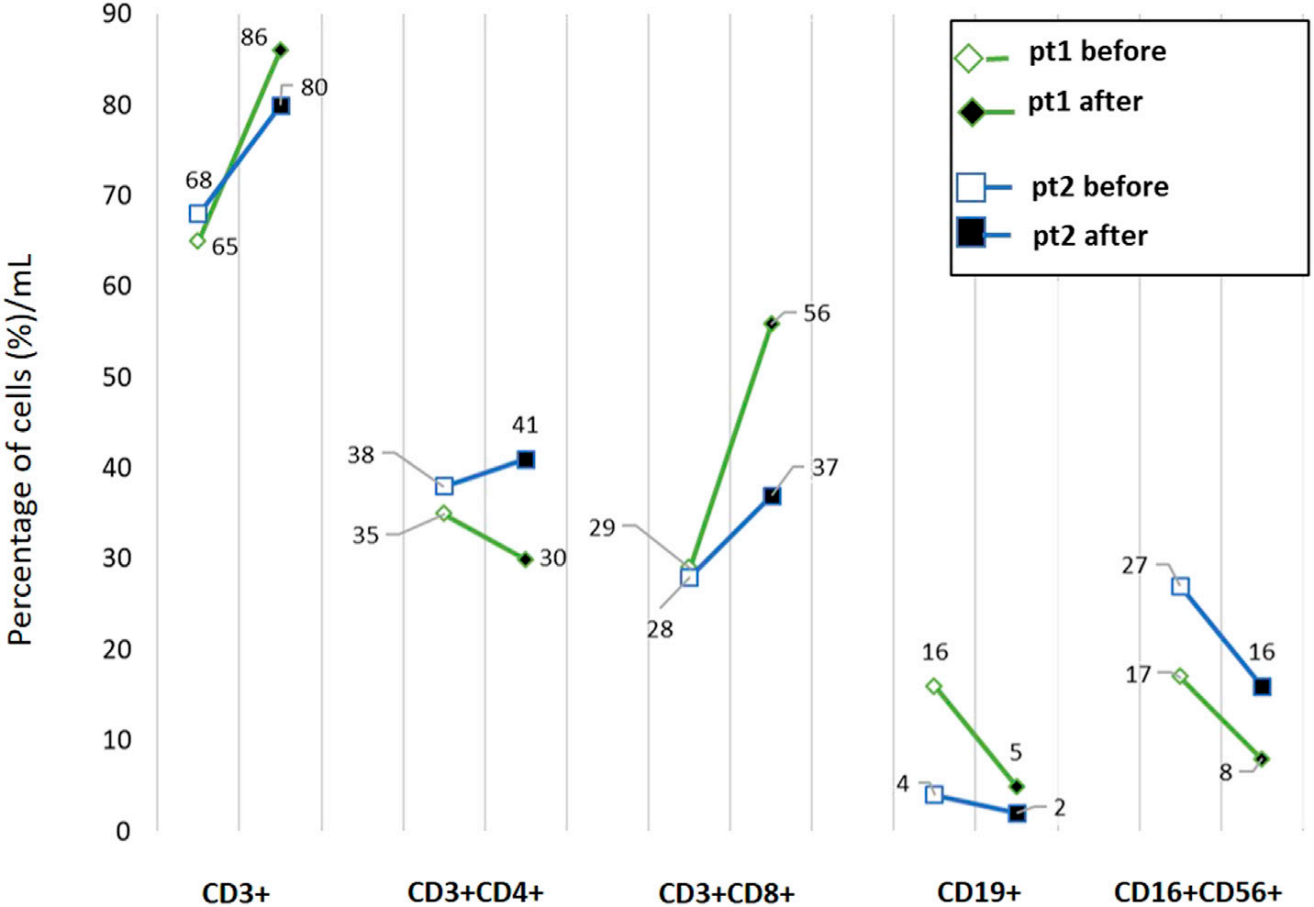
Flow cytometric profiling of peripheral blood lymphocyte subsets in two patients enrolled in the study. Whole blood samples were analyzed by multiparameter flow cytometry to quantify the frequencies of CD3^+^ T cells, CD3^+^CD4^+^ helper T cells, CD3^+^CD8^+^ cytotoxic T cells, CD19^+^ B lymphocytes and natural killer (CD3^−^CD16^+^CD56^+^) cells. Samples were collected at the beginning of the treatment (before) and after the appearance of irAE (after). pt: patient.

As reported in Figure 1, a reduction in B cells and NK cell frequency was observed in both patients whereas an almost two-fold increase in the percentage of circulating CD3^+^CD8^+^ T cells was detected in patient 1. In this patient, the total amount of lymphocytes dramatically decreased with the onset of the irAE, as was also evident in the blood analyses.

### 2.3 Case 3

#### 2.3.1 Patient information and clinical findings

The third patient was an 84-year-old man with a diagnosis of stage III melanoma of the right shoulder on February 2023. He underwent resection of the primary tumor with subsequent wide local excision and sentinel lymph nodes biopsy. BRAF V600 was wild type, but several mutations in the exon 11 were detected, in association to N-RAS Q61X mutation.

#### 2.3.2 Timeline

The patient began adjuvant treatment with nivolumab (240 mg) every 2 weeks in March 2023. Seven months later, in October 2023, the patient reported asthenia, muscle cramps, dyspnea with mild exertion, and edema in the lower extremities. Blood tests revealed the following: thyroid-stimulating hormone (TSH), 60 µUI/mL; reduced hemoglobin and white blood cell counts, 6.6 g/dL and 2.9 × 10^3^ µL, respectively; increased total and indirect bilirubin levels, 2.22 and 1.59 mg/dL, respectively; reduced haptoglobin levels, 29 mg/dL; normal iron levels; increased ferritin levels; increased LDH levels, 600 U/L; normal vitamin B12 levels; reduced folate levels; increased erythropoietin levels, 30 mU/mL. A CT scan performed in November 2023 excluded disease progression.

#### 2.3.3 Outcomes and follow-up

The patient received three blood transfusions, was started on steroid therapy at a dosage of 1 mg/kg. Folic acid and vitamin B12 were supplemented, and levothyroxine therapy was started at a dosage of 50 mg daily, which was increased progressively. Thirteen days later, the patient was discharged in good clinical condition with improved biochemical parameters. Immunotherapy was discontinued, and the patient has survived for 23 months without disease progression.

### 2.4 Case 4

#### 2.4.1 Patient information and clinical findings

The fourth patient was a 78-year-old woman, with stage IIIc melanoma in the left scapular region. A BRAF mutation was detected at codon 601 (K601E). The patient had a positive history of allergic reactions to several antibiotics and anti-inflammatory medications. She had a good performance status, and her hemoglobin level was 12 g/dL at the beginning of adjuvant immunotherapy with pembrolizumab (200 mg every 21 days).

#### 2.4.2 Timeline

After two cycles of ICIs, the patient complained of dyspnea with mild exertion and asthenia (grade 2). Blood tests revealed the following results: hemoglobin, 11.4 g/dL; red blood cells (RBCs), 1.38 × 10^6^ μL; hematocrit (HCT), 13.3% (normal range, 38–46); and elevated total bilirubin and LDH levels (1.8 and 263 U/L, respectively). A CT scan performed 1 month after the initial signs of anemia (October 2024) excluded disease progression. Iron and vitamin B12 levels were within the normal range, with increased ferritin and reduced haptoglobin levels (<8). A positive Direct Antiglobulin Test (DAT) showed the presence of IgM cold autoantibodies that attack the patient’s red blood cells specifically at temperatures below 37 °C, causing agglutination. The titer was 128 at 4 °C.

#### 2.4.3 Outcomes and follow-up

The patient underwent multiple hematologic consultations that confirmed the diagnosis of AIHA. The patient was started on oral steroids (prednisone 0.5 mg/kg) which improved and recovered over a period of 2 months. Immunotherapy was then discontinued. Eight months later, the disease progressed to the lungs, liver, and bones. The patient started taking anti-BRAF and MEK inhibitors, which they are still taking.

### 2.5 Case 5

#### 2.5.1 Patient information and clinical findings

The fifth patient was a 78-year-old man, with a diagnosis of cutaneous squamous cell carcinoma, locally advanced with axillary lymph node metastases. The patient had a medical history of multiple myeloma, in follow-up. After consulting with the hematologist in July 2024, the patient began immunotherapy with cemiplimab, 350 mg every 3 weeks. The patient achieved a complete response after four cycles of therapy.

#### 2.5.2 Timeline

During the visit for the sixth cycle in November 2024, the patient complained of diarrhea (three to four stools daily) for 14 days. Platelet count was 60 × 10^3^ μL versus the baseline of 200 × 10^3^ μL. A blood count was performed using both EDTA and sodium citrate with no difference observed. At the initial evaluation, the hematologist excluded myeloma progression.

#### 2.5.3 Outcomes and follow-up

Five days later, due to a further reduction in platelets (35 × 103 µL), prednisone was initiated at a dose of 1 mg/kg. One week later, however, the platelet count had decreased to 20 × 10^3^ µL, while the hemoglobin and white blood cell counts had decreased to 8 g/dL and 2,210, respectively. The LDH level increased to 362 U/L. A PET/CT performed on January 2025 excluded disease progression.

The hematologist performed a bone marrow biopsy, which revealed myelodysplastic syndrome (MDS) with a slight increase in the blast cells. None of the tested genetic abnormalities (monosomy 5/del (5q), monosomy 7/del (7q), deletion 20q, and trisomy 8) were present. FISH analysis showed no chromosomal alterations in the 200 cells examined for each probe. The steroid dosage was reduced, and the patient began follow-up for squamous cell carcinoma. At the last follow-up in our clinic, the patient was disease free of squamous cell carcinoma. However, due to the MDS, the patient passed away in May 2025.

## 3 Discussion

In this case series, we present a report on hematologic toxicities associated with ICIs in five skin cancer patients. During a 3-year period at our institute, severe hematologic toxicity occurred in 2.1% of patients (five out of 237). The median age at diagnosis was 78 years, and the patients were in both the metastatic and adjuvant settings. According to the literature, the onset of toxicity is reported from the third to the eighth decades of life (Boegeholz et al., 2020), with a median age of diagnosis ranging from 59 to 66 years and differing by hematologic toxicity. This is lower than the median age of our patients (Barnes et al., 2021; Davis et al., 2019).

In our series, the median time to onset was early: 3.5 months. According to the literature, the median time to onset of anemia ranges from 2.5 to 18 months, with a median of 4 weeks (Delanoy et al., 2019). Regarding neutropenia, the median time to onset was 10.5 weeks after the first ICI administration, and neutrophil counts normalized after a median duration of 13 days (Boegeholz et al., 2020; Patel and Pai, 2020). Despite the use of high doses of steroids, intravenous immunoglobulin (IVIG), growth factors, and antibiotics, death was reported in 41% of patients. Similarly, in our series, the patient who experienced neutropenia died 1 month after the onset of hematologic toxicity. This toxicity occurred concomitantly with disease progression and proved fatal quickly.

Most of our patients (80%) were male. Another case series of 35 patients (Delanoy et al., 2019) also reported this gender prevalence in the development of hematologic irAE, in which men outnumbered women by a factor of 1.5. Four out of five of our patients had melanoma, which is more prevalent in men than in women (Bellenghi et al., 2020). This may explain the prevalence of hematologic irAEs that we observed. However, another study reported a lower prevalence of hematologic irAEs in men than in women, in contrast to other irAEs, such as dermatological or gastrointestinal irAEs, which are more prevalent in women than in men (Auch et al., 2025).

Although oncological guidelines, such as those from the European Society for Medical Oncology (ESMO), address hematologic toxicities (including AIHA) as part of irAEs management, they lack detailed flowcharts or differentiated approaches for warm AIHA and cold agglutinin disease (Haanen et al., 2022; Barcellini and Fattizzo, 2023). Integrating these guidelines would help to better characterize these toxicities and ensure their appropriate and effective management.

Hematologic irAEs were not related to the response to ICIs. In fact, they occurred in patients who experienced disease progression, as well as in those who had partial or complete responses. None of our patients restarted ICIs after discontinuation, however, 60% are still alive. Only one of the surviving patients was disease-free at the last follow-up. Progression was managed with targeted therapy in one case and local treatment in another. Since hematologic irAEs are very rare, recurrence rates after resumption could not be clearly determined. According to the literature, rechallenge with the same ICI has been attempted in 20% of patients, with irAEs occurring in 43% of cases (Delanoy et al., 2019).

The pathogenesis of hematologic irAEs is not fully understood. Some authors suggest that it involves an overactivation of T lymphocytes, autoantibody production, and/or cytokine dysregulation (Kroll et al., 2022).

For our patients, we speculate that the following could be warning signs of subsequent hematologic irAE onset: previous diarrhea due to ICIs (patients 1 and 2); asthenia (patients 3 and 4); a sudden increase in LDH values in the absence of verified disease progression via imaging data, such as a CT scan (patients 2, 3, 4, and 5).

In case 5, the role of cemiplimab in the development of MDS is unknown. However, the temporal link may suggest a contribution. The temporal link may suggest a contribution. Several years prior, this patient had multiple myeloma, which was in remission at the time of ICI treatment. This case is peculiar because the MDS appeared after a few cycles of ICIs, despite a complete response to the squamous cell carcinoma. The Naranjo Adverse Drug Reaction Probability Scale was applied, and the result was POSSIBLE.

MDS is common in older adults and is associated with additional risk factors, such as prior exposure to radiotherapy or chemotherapy (particularly alkylating agents) and genetic susceptibility. Our patient had multiple risk factors: advanced age and a history of radiotherapy for squamous cell carcinoma, as well as chemotherapy for myeloma several years earlier. However, the specific chemotherapy regimen administered previously is unknown. Furthermore, recent papers have reported on patients with solid tumors who developed hematologic neoplasia following ICIs as an additional risk factor. Van Eijs et al. (van Eijs et al., 2023) hypothesize that PD-1 blockade may accelerate the progression of overt myeloid malignancies and contribute to the clonal selection of malignantly transformed progenitors. A previous report described a patient with a concomitant medical history of B-cell chronic lymphocytic leukemia and advanced squamous cell carcinoma who experienced hematologic irAEs (Schwab et al., 2016). In our case 5, the patient had a partial response to squamous cell carcinoma treatment with ICIs. However, nivolumab was withheld due to hematologic toxicity and was not restarted. The B-cell chronic lymphocytic leukemia was also stable at the time of the event (Schwab et al., 2016). Patient 4 had a history of allergic reactions to several antibiotics and anti-inflammatory medications. However, no correlation has been described between multiple allergies and the risk of irAEs for ICI therapy. Despite their different mechanisms of action, we cannot rule out the presence of interfering causal factors.

Currently, there are no approved biomarkers available for predicting the risk of irAEs. The roles of specific HLA types, single-nucleotide polymorphisms, microRNAs, cytokines, and microbiome composition have been investigated and correlated with toxicities. However, none have been adopted in clinical practice (Goodman et al., 2023). Lim et al. developed the CYTOX score, which is a predictive tool for severe irAEs in melanoma patients treated with ICIs. This score includes pro-inflammatory cytokines and may facilitate the early management of severe, potentially life-threatening irAEs, such as hematologic ones (Johnson and Balko, 2019; Lim et al., 2019). However, we could not evaluate this score in our patients. Previous studies have not correlated specific circulating immune cell subsets with hematologic toxicities in patients treated with ICIs. We collected blood samples from two melanoma patients at the beginning of ICI treatment and at the onset of hematologic irAEs and performed cytofluorimetric analyses. However, flow cytometry analysis was possible only for two patients, which limits the ability to generalize the results. We found that alterations in the percentage and absolute number of circulating immune cells can occur during ICI treatment. We observed a reduction in the frequency of B cells and NK cells. Notably, a high frequency of circulating B cells appears to prevent ICI-induced irAEs in melanoma (Willsmore et al., 2025). In previous studies, we did not observe a difference in circulating B and NK cells at the time of vitiligo onset, a cutaneous irAE frequently observed in melanoma patients undergoing ICI therapy (Carbone et al., 2023). Therefore, it is possible that hematologic toxicities induce specific changes in different circulating immune cell subsets. In patient 1, we also observed an increase in the percentage of CD8^+^ T cells above normal values with the onset of the hematologic irAE. This result likely reflects a state of systemic immune hyperactivation rather than an effective antitumor immunity, suggesting that highly activated CD8^+^ T cells contributed to widespread tissue damage and hematopoietic suppression leading to fatal outcome in this patient.

We also found that three of our five patients (60%) had N-RAS or rare BRAF mutations. Data were incomplete or unavailable for the remaining two patients. Thus far, no studies have correlated hematologic toxicities with mutational status. Since RAS pathway mutations have been detected in approximately half of hematopoietic malignancies, we hypothesize that N-RAS mutations may contribute to hematologic irAEs. Further validation studies are needed to confirm this hypothesis.

The main limitations of this study are its retrospective nature, the potential under-reporting of irAEs, and the small number of cases. Additionally, hematologic data, such as reticulocyte count and DAT, were not always available. Only patient 4 underwent thorough characterization, including reticulocyte count and DAT. Coombs test was done only to patient 2. No data on peripheral smears or infectious workups were available for our patients. Therefore, we could not draw any definitive conclusions. However, we can raise concerns about the possible development of hematologic irAEs in patients: 1) those with concomitant hematologic disease, 2) those with a history of multiple allergies, 3) those who previously experienced diarrhea as an irAE, and 4) those with N-RAS or uncommon BRAF mutations. We recommend paying closer attention to these patients and performing a DAT/Coombs test, as well as analyzing circulating immune cell subsets if possible. Despite the small number of patients, our work suggests the existence of potential biomarkers associated with these rare and severe events. Our study also emphasizes the need for detailed management algorithms in oncology to improve detection and treatment, as is already established for hematologic diseases. Furthermore, our study posits that identifying early warning signs could raise clinical suspicion and enable prompt recognition.

## 4 Patient perspective

Hematologic irAEs are rare but life-threatening complications that often cause interruption of ICI therapy. Because there are no biomarkers that can predict this toxicity in the adjuvant or metastatic setting, we use available clinical and laboratory data to identify warning signs of the event. We highlight a few clinical features that could help identify at-risk patients who should undergo further analysis, such as a Coombs test.

## Data Availability

The raw data supporting the conclusions of this article will be made available by the authors, without undue reservation.

## References

[B1] Auch L. A. M. Sieber C. Lehnick D. Hug B. L. (2025). Adverse drug events of immune checkpoint inhibitors - a retrospective, descriptive real-world data analysis. BMC Cancer 25 (1), 1303. 10.1186/s12885-025-14733-5 40790180 PMC12337401

[B2] Barcellini W. Fattizzo B. (2023). How I treat warm autoimmune hemolytic anemia. Blood 137 (10), 1283–1294. 10.1182/blood.2022019024 33512406

[B3] Barnes R. Crnp M. N. Aocn A. N. P.-B. C. B. Zawislak MPAS Pa-C C. Wong P. A.-C. V. (2021). Management of hematologic adverse events associated with immune checkpoint inhibitors. J. Adv. Pract. Oncol. 12 (4), 392–404. 10.6004/jadpro.2021.12.4.4 34123476 PMC8163252

[B16] Bellenghi M. Puglisi R. Pontecorvi G. De Feo A. Carè A. Mattia G. (2020). Sex and gender disparities in melanoma. Cancers (Basel) 12 (7). 10.3390/cancers12071819 32645881 PMC7408637

[B17] Boegeholz J. Brueggen C. S. Pauli C. Dimitriou F. Haralambieva E. Dummer R. (2020). Challenges in diagnosis and management of neutropenia upon exposure to immune-checkpoint inhibitors: Meta-analysis of a rare immune-related adverse side effect. BMC Cancer 20 (1). 10.1186/s12885-020-06763-y 32290812 PMC7155336

[B4] Carbone M. L. Capone A. Guercio M. Reddel S. Silvestris D. A. Lulli D. (2023). Insight into immune profile associated with vitiligo onset and anti-tumoral response in melanoma patients receiving anti-PD-1 immunotherapy. Front. Immunol. 14, 1197630. 10.3389/fimmu.2023.1197630 37680638 PMC10482109

[B5] Carlino M. S. Larkin J. Long G. V. (2021). Immune checkpoint inhibitors in melanoma. Lancet 398 (Issue 10304), 1002–1014. 10.1016/S0140-6736(21)01206-X 34509219

[B6] Darnell E. P. Mooradian M. J. Baruch E. N. Yilmaz M. Reynolds K. L. (2020). Immune-related adverse events (irAEs): diagnosis, management, and clinical pearls. Curr. Oncol. Rep. 22 (Issue 4), 39. 10.1007/s11912-020-0897-9 32200442

[B7] Davis E. J. Salem J.-E. Young A. Green J. R. Ferrell P. B. Ancell K. K. (2019). Hematologic complications of immune checkpoint inhibitors. Oncol. 24 (5), 584–588. 10.1634/theoncologist.2018-0574 30819785 PMC6516131

[B8] Delanoy N. Michot J. M. Comont T. Kramkimel N. Lazarovici J. Dupont R. (2019). Haematological immune-related adverse events induced by anti-PD-1 or anti-PD-L1 immunotherapy: a descriptive observational study. Lancet Haematol. 6 (1), e48–e57. 10.1016/S2352-3026(18)30175-3 30528137

[B18] Di Lorenzo G. Michele A. Silvana L. Bilancia D. Di Trolio R. Iuliucci M. R. (2024). A retrospective study of cemiplimab effectiveness in elderly patients with squamous cell carcinoma of the skin: insights from a Real-Life Scenario. Oncol. ther. 12 (1). 10.1007/s40487-023-00256-1 38112965 PMC10881452

[B19] Goodman R. S. Jung S. Balko J. M. Johnson D. B. (2023). Biomarkers of immune checkpoint inhibitor response and toxicity: Challenges and opportunities. Immunol. Rev. 318 (1). 10.1111/imr.13249 37470280 PMC10528475

[B9] Haanen J. Obeid M. Spain L. Carbonnel F. Wang Y. Robert C. (2022). Management of toxicities from immunotherapy: ESMO clinical practice guideline for diagnosis, treatment and follow-up. Ann. Oncol. 33 (12), 1217, 1238. 10.1016/j.annonc.2022.10.001 36270461

[B20] Johnson D. B. Balko J. M. (2019). Biomarkers for immunotherapy toxicity: Are cytokines the answer?. Cancer Res. 25 (5). 10.1158/1078-0432.CCR-18-3858 30587548 PMC6397678

[B21] Keam S. Turner N. Kugeratski F. G. Rico R. Colunga-Minutti J. Poojary R. (2024). Toxicity in the era of immune checkpoint inhibitor therapy. Front Immunol. 15. 10.3389/fimmu.2024.1447021 39247203 PMC11377343

[B10] Kramer R. Zaremba A. Moreira A. Ugurel S. Johnson D. B. Hassel J. C. (2021). Hematological immune related adverse events after treatment with immune checkpoint inhibitors. Eur. J. Cancer 147, 170–181. 10.1016/j.ejca.2021.01.013 33706206

[B11] Kroll M. H. Rojas-Hernandez C. Yee C. (2022). Hematologic complications of immune checkpoint inhibitors. Blood 139 (25), 3594–3604. 10.1182/blood.2020009016 34610113 PMC9227102

[B22] Lim S. Y. Lee J. H. Gide T. N. Menzies A. M. Guminski A. Carlino M. S. (2019). Circulating cytokines predict immune-related toxicity in melanoma patients receiving anti-PD-1–based immunotherapy. Cancer Res. 25 (5). 10.1158/1078-0432.CCR-18-2795 30409824

[B12] Michot J. M. Lazarovici J. Tieu A. Champiat S. Voisin A. L. Ebbo M. (2019). Haematological immune-related adverse events with immune checkpoint inhibitors, how to manage? Eur. J. Cancer 122, 72–90. 10.1016/j.ejca.2019.07.014 31634647

[B23] Omar N. E. El-Fass K. A. Abushouk A. I. Elbaghdady N. Barakat A. E. M. Noreldin A. E. (2020). Diagnosis and Management of Hematological Adverse Events Induced by Immune Checkpoint Inhibitors: A Systematic Review. Front. Immunol. 11. 10.3389/fimmu.2020.01354 33193289 PMC7640759

[B24] Patel R. Pai L. (2020). Immunotherapy-induced severe neutropenia with neurotoxicity: A case of a 75-year-old woman with ulcerative colitis diagnosed with melanoma. J. Oncol. Pharm. Pract. 26 (3). 10.1177/1078155219862341 31315551

[B13] Schwab K. S. Heine A. Weimann T. Kristiansen G. Brossart P. (2016). Development of hemolytic anemia in a nivolumab-treated patient with refractory metastatic squamous cell skin cancer and chronic lymphatic leukemia. Case Rep. Oncol. 9 (2), 373–378. 10.1159/000447508 27462240 PMC4939691

[B14] van Eijs M. J. M. van der Wagen L. E. Mous R. Leguit R. J. van de Corput L. van Lindert A. S. R. (2023). Hematologic malignancies following immune checkpoint inhibition for solid tumors. Cancer Immunol. Immunother. 72 (1), 249–255. 10.1007/s00262-022-03230-4 35691988 PMC9188911

[B15] Willsmore Z. N. Booth L. Patel A. Di Meo A. Prassas I. Chauhan J. (2025). Circulating immunoregulatory B cell and autoreactive antibody profiles predict lack of toxicity to anti-PD-1 checkpoint inhibitor treatment in advanced melanoma. J. Immunother. Cancer 13 (5), e011682. 10.1136/jitc-2025-011682 40449958 PMC12142029

