# Supplementary material for: Hematologic immune-related adverse events in skin cancer patients treated with immune checkpoint inhibitors: a case series
Source: Front Pharmacol. 2026 Jan 6;16:1717727. doi: 10.3389/fphar.2025.1717727 (PMC12816168; doi:10.3389/fphar.2025.1717727)
Supplement: Supplementary file 1 [file Image1.pdf]

Supplementary Figure 1

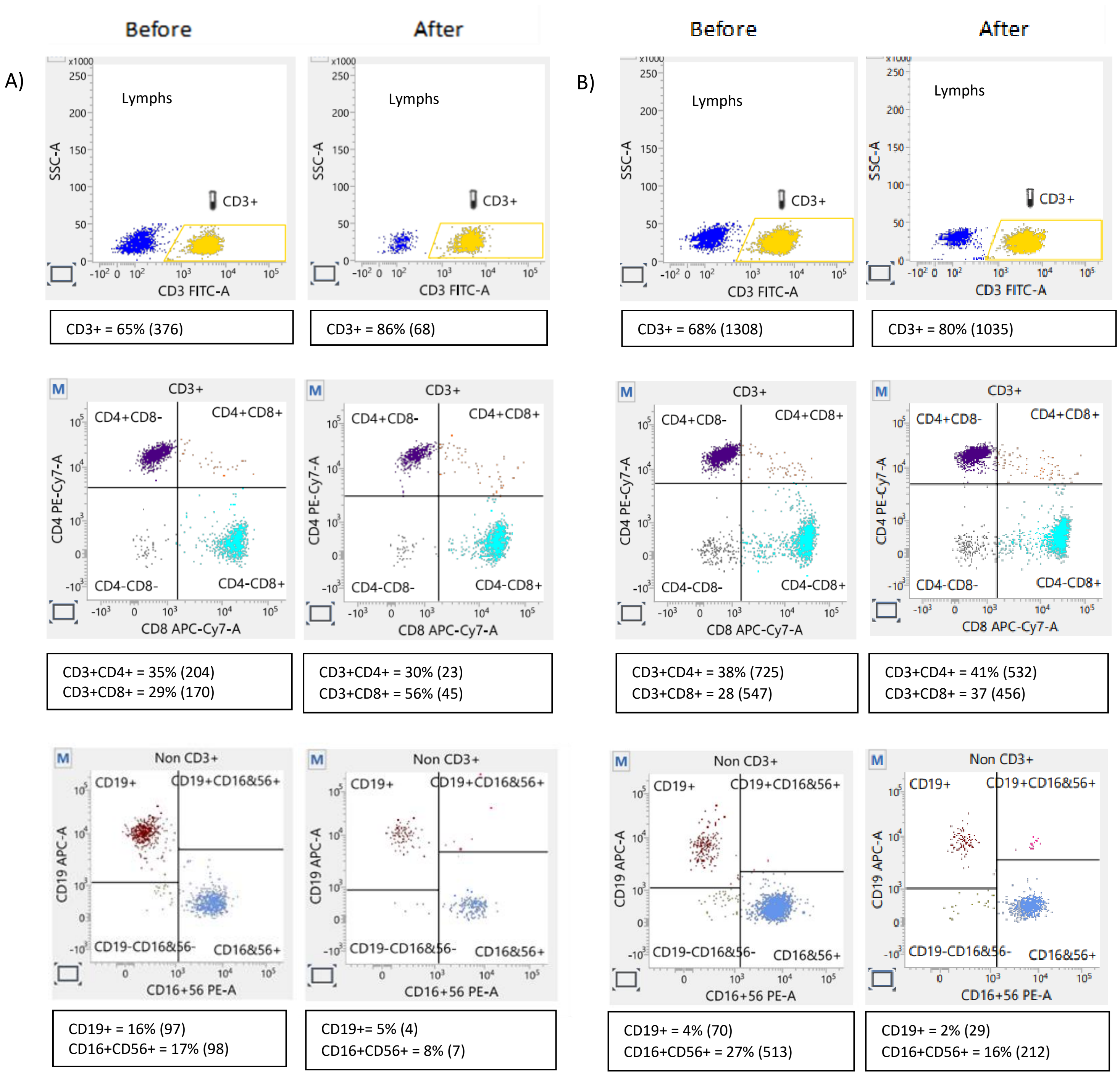

Immunophenotyping of peripheral blood by multiparametric flow cytometry. The analyses was done in Pt 1 (A) and in Pt 2 (B) blood samples, at the beginning of the treatment (before) and after the appearance of the hematologic irAE (after). Dot plot derived from laboratory report template are reported and relative values in both percentage (%) and absolute number (n) are indicated below each plot. Lymphs: lymphocytes.

MATERIALS AND METHODS

Cytofluorimetric analysis. Whole blood was stained with BD Multitest 6-color TBNK kit (Becton Dickinson) in a single tube lyse–no-wash assay to simultaneously measure T, B, and NK cell subsets and calculate absolute counts directly by using BD TruCOUNT tubes with microsphere. Samples were prepared by mixing 50 µl of EDTA whole blood with 20 µl of labelled antibody mixture (CD3 FITC, CD16+CD56 PE, CD45 PerCP-Cy5.5, CD4 PE-Cy7, CD19 APC, CD8 APC-Cy7) and incubated for 20 minutes at room temperature in the dark. Then, 500 µl of lysing solution was added and, after 10 minutes of incubation, samples were analyzed on the BD FACSLyric flow cytometry system (three lasers - 10 color configuration). The CD45/SSC gating strategy was used for identification of lymphocytes and for the subsequent evaluation of positive cells for each antigen. The following gating hierarchy was applied: within the lymphocyte gate, a dot plot CD3/SSC identified total T lymphocytes and a subsequent quadrant gate on CD3+ cells defined T cells subset as CD3+CD4+CD8- helper T cells and CD3+CD8+CD4- cytotoxic T cells. The CD19/CD16 CD56 dot plot identified respectively total B and NK cells with a quadrant gate within the CD3 negative population.
